# Are Measles-Mumps-Rubella (MMR) Antibodies Friends or Foes for Covid-19 Disease?

**DOI:** 10.1007/s00005-023-00680-1

**Published:** 2023-06-21

**Authors:** Azza Salamony, Yara Shamikh, Khaled Amer, Tarek Elnagdy, Mostafa Elnakib, Abd Allah Yehia, Wael Hassan, Maha Abdelsalam

**Affiliations:** 1 Egypt Centre for Research and Regenerative Medicine, ECRRM, Cairo, 11517 Egypt; 2 Microbiology and Immunology, Central Public Health Laboratories, CPHL, Ministry of Health, Cairo, 11613 Egypt; 3 Clinical Pathology Department, Faculty of Medicine, Immunology Unit, Mansoura University, Mansoura, 35516 Egypt

**Keywords:** Antibodies titre, COVID-19, ELISA, MMR

## Abstract

Many factors have been implicated in the pathogenesis and severity of COVID-19 pandemic. A wide variation in the susceptibility for SARS-CoV-2 infection among different population, gender and age has been observed. Multiple studies investigated the relationship between the antibody’s titre of previously vaccinated individuals and the susceptibility of coronavirus infection, to find a rapid effective therapy for this pandemic. This study focused on the association between measles-mumps-rubella (MMR) antibodies titre and the severity of COVID-19 infection. We aimed to investigate the correlation between the antibody’s titre of MMR and the SARS-CoV-2 infection susceptibility and disease severity, in a cohort of COVID-19 Egyptian patients, compared to a control group. MMR antibody titre was measured using enzyme Linked Immune Sorbent Assay; (ELISA) for 136 COVID-19 patients and 44 healthy individuals, as control group. There were high levels of measles and mumps antibodies titer in the deteriorating cases, which could not protect from SARS-CoV-2 infection. However, the rubella antibodies might protect from SARS-CoV-2 infection, but once the infection occurs, it may aggravate the risk of case deterioration. MMR antibodies could be used as a guideline for COVID-19 symptom-severity and, in turn, may be considered as an economic prognostic marker used for early protection from multiple autoimmune organ failure.

## Introduction

The measles virus (MV) is an aerosol-borne, highly transmissible, contagious, single-stranded, negative-sense, enveloped, non-segmented and human-specific RNA virus, of the genus *Morbillivirus*, within the family *Paramyxoviridae* (Rima et al. 2019). In 1963, a wild-type, attenuated in vitro infected human and chicken cells was licensed as a safe and potent vaccine without any indications of safety and efficacy issue (Griffin 2018; Holzmann et al. 2016).

The mumps virus is another negative-sense, enveloped, non-segmented and human-specific RNA virus within the family *Paramyxoviridae* also (Rima et al. 2019), which was first described in the fifth century BC, by Hippocrates in his first book of Epidemics; then its etiology was demonstrated in the 1930s (Johnson and Goodpasture 1934, 1935). It is a self-limiting infection, with complete recovery within a few weeks of symptoms’ first onset (Rubin et al. 2015). The mumps vaccine, Mumpsvax®, was licensed in 1967 (Young et al. 1967).

The rubella virus is a positive-stranded sense RNA, the sole member of the genus *Rubivirus*, in the family of *Togaviridae*, and it has human as its only natural host (Sakata and Mori 2014). The rubella vaccine, Meruvax®, was developed in Wistar Institute, in 1967, in a human lung cell and licensed in 1969 (Plotkin et al. 1967).

As mentioned above, each virus had its own licensed vaccine during the 1960s (Goodson and Seward 2015); then in 1971, a combination of the three viruses’ vaccine was invented by Maurice Hilleman and named MMR (measles-mumps-rubella) vaccine (Newman 2005), which was then, licensed for usage by Merck & Co™. It is prescribed in two doses for children 9–15 months of age followed by another dose at 25 months to 6 year olds (World Health Organization 2019).

The MMR vaccine has been mandatory in Egypt for infants since 1999, with two routine doses, as prescribed worldwide, targeting infants and 6–16-year-old children. Then a nation-wide measles-rubella immunization campaign was conducted in 2008–2009 targeting children and young adults between the ages of 2–20 years, and achieved coverage of > 95% (El Sayed et al. 2011). The effect of the vaccine can be assessed using enzyme immunoassay examination to measure the immunoglobulin G (IgG) antibody in serum samples of previously vaccinated candidates to determine the existence of immunity against these viruses (Sasaki et al. 2019).

On the other hand, since the early 2000’s, many novel coronaviruses (CoV) emerged causing severe diseases. They belong to the family *Coronaviridae,* order *Nidovirales* (Weiss and Leibowitz 2011). The first emerged disease was the severe acute respiratory syndrome (SARS) caused by the coronavirus SARS-CoV, which occurred at the end of winter 2003 in China and 29 other territories, with more than 8000 infected cases and lasted only for less than 1 year (Peeri et al. 2020; Yang et al. 2020).

The second disease that appeared was the Middle East respiratory syndrome (MERS) caused by the Middle East respiratory syndrome virus, MERS-CoV, in June 2012, in Jeddah, Saudi Arabia (Zaki et al. 2012), leading to limited number of infected cases (Sayed et al. 2020) and not more than 858 known deaths (Peeri et al. 2020; Yang et al. 2020).

The last emerged disease was caused by a new strain of coronavirus, known as the severe acute respiratory syndrome coronavirus-2 (SARS-CoV-2) which first appeared in Wuhan, China, in 2019, and spread rapidly worldwide, causing COVID-19 disease and announced as a pandemic, by the World Health Organization, in March 2020 (Ludwig and Zarbock 2020; Orfali et al. 2021).

Since then, multiple studies have been published, to understand the course of infection of this disease, and even to relate it with many other influencing factors, such as its association with the ABO blood-type to develop specific anti-histo-blood group antibodies for its treatment as co-therapy (Shamikh et al. 2021).

Many trials had been executed to produce protective vaccines against these newly emerged viruses. In 2008, a new recombinant bivalent live attenuated measles vaccine (rMV) was generated, including parts of the nucleo-capsid protein, (N), or spike glycoprotein (S), of the newly emerged SARS-CoV. This vaccine produced high neutralizing antibodies against both SARS-CoV and MV (Liniger et al. 2008). High neutralizing antibody titre was discovered in 2008, from the rMV vaccinated animals, after SARS-intranasal challenges, even after changing the expressed part of the SARS-CoV to be its membrane-anchored S-protein or its secreted soluble ecto-domain (Escriou et al. 2014). The same recombinant technology was used in 2012, but instead of S-SARS-CoV protein, it used S-MERS protein from the newly emerged MERS virus, back then. This rMV-MERS vaccine gave the vaccinated animals high immunity both cellular and humoral (Malczyk et al. 2015).

Although SARS-CoV-2 pandemic crisis has a little dissipation in some countries, it has been discovered that the countries with lower death rates and lower severity of symptoms from COVID-19 infection are those with large-scale MMR vaccination campaigns (Gold et al. 2020). Some other researchers have been more specific to propose thatthe MMR vaccine, could induce self-protection against COVID-19 infection or even decrease its severity, prevalence and mortality rate (Anbarasu et al. 2020; Elhusseiny et al. 2020; Meenakshisundaram et al. 2020; Sidiq et al. 2020).

Many theories proposed a reasonable hypothesis for this finding. A recent systematic review, published in Iran by Taheri Soodejani et al. (2021)on 169 published documents, correlates the lower rate of infection in children to their higher level of immunity, gained by their routine vaccination (Anbarasu et al. 2020). It also correlates the similarity between the measles, mumps and rubella viruses’ spike glycoprotein structure to the SARS-CoV-2 structure to be 32%, 31%, 33% similarity, respectively, proposing that the immunity gained from receiving the MMR vaccine is the reason for the lower infection rate (Ashford et al. 2021; Hanker 2020; Saad and Elsalamony 2020).

Another hypothesis made a correlation between induce interferons (IFNs) and activated natural killer (NK) cells, produced from receiving the MMR vaccine, which increase the natural immunity against SARS-CoV-2 (Anbarasu et al. 2020). The immune cells have the power to enhance resistance upon re-infection with related, or unrelated viruses in a discovered character of the bone marrow cells, which is called trained immunity (Netea et al. 2016). They also produce pre-existing antibodies which in turn provide pre-existing immunity due to their cross-reactivity (Marakasova and Baranova 2021).

Another hypothesis suggests that the milder course of infection in children may be due to some parts of SARS-CoV-2 S-glycoprotein, which could interfere with some parts of the measles and rubella vaccine, which are the fusion protein F1 and the membrane glycoprotein E1, respectively (Fidel and Noverr 2020).

Other theories related to the immune system reaction with the severity of COVID-19 infection. In COVID-19, severe cases that were admitted to the Intensive Care Unit (ICU), there was a reported phenomenon known as the cytokine storm. In this phenomenon, one of the first line-immune cells in the lungs, are macrophages, which were involved in the pathogenesis of the life-threatening acute respiratory distress syndrome (Mehta et al. 2020). These macrophages were over-activated, causing a hyper-stimulation in the patients’ immune system (Huang et al. 2020) and increased the secreted levels of cytokines, as an inflammatory mediator. Furthermore, the produced cytokines caused the inflammation to increase (Russell et al. 2020).

Another study discovered anti-nuclear autoantibodies which are autoimmune indicators and lupus anti-coagulants, in patients admitted to the hospital with SARS-CoV-2 pneumonia (Gazzaruso et al. 2020). Also, in a case-based review, high anti-Sjögren’s-syndrome-related antigen-A autoantibody titre has been discovered as autoimmune response in COVID-19 patients (Fujii et al. 2020).

High autoantibodies level as anti-cardiolipin and/or anti-β2 glycoprotein-1, together with high level of factor VIII, was detected by antiphospholipid profile in severe COVID-19 patients, which lead to hyper-coagulation (Zhang et al. 2020). Other types of autoantibodies are the anti-melanoma differentiation-associated gene 5 and the antibodies against type-I IFNs, which were, also, detected in severe COVID-19 patients (Bastard et al. 2020).

Thus, in our study, we explored the association between the antibodies of the MMR vaccine, and the newly discovered SARS-CoV-2, by determining the antibodies titre for these three viruses separately in COVID-19-positive patients and compared them to the antibody’s titre in COVID-19-negative patients.

## Materials and Methods

### Patients

There were 180 individuals involved in this study. They were admitted to the *Fever Hospitals*, during November and December 2021. The study included both sexes, 35 females and 145 males, with different age groups, but all of them were above 18 years of age, and they exhibited all different symptoms and outcomes. The candidates were divided into two main groups. The first group was the control group, which was composed of 44 individuals with negative-PCR test for SARS-CoV-2, despite being in close contact to positive COVID-19 confirmed cases. The second group, included 136 patients with a positive-PCR test for SARS-CoV-2 and further has been subdivided into three main subgroups, according to the severity of their infection. The first subcategory had 58 patients with mild COVID-19 symptoms and were not hospitalized, while the second subcategory had 45 patients presented with moderate symptoms and were hospitalized, but had not been admitted to the ICU, and the last subgroup had 33 patients with severe symptoms and they had been admitted to the ICU. We did not classify level of deterioration, but all deteriorated patients admitted to ICU were included.

### Sample Collection

The samples in our study were 2 ml of serum blood samples for measles, mumps and rubella tests from each individual/patient. The samples were taken at diagnosis after appearance of symptoms and positive COVID-19 PCR test. Patients had different degree of severity at time of diagnosis.

### Diagnostic Kits

#### Measles Kits

We used Euro-immune-anti-Measles virus ELISA IgG kits. The kits were used as prescribed by the manufacturer, where all kits containing calibrators, controls, enzyme conjugate and stop solution were ready to use except for the samples, which were diluted by sample buffer in the ratio 1:100 and the wash solution which was diluted 10 ml wash buffer + 990 ml distilled water.

#### Procedures

The procedures were followed as prescribed also by the manufacturer, from sampling, adding controls and calibrators, incubations, washing, enzyme conjugation, chromogen substrate addition, then stopping any further reactions for the plates to be measured for its colour intensity at 450 nm wave length and 620–650 nm reference wave length within 30 min.

### Plates are Validated by the Reading of the following:

- Cal 1 > 0.7 OD, Cal 2 > 0.994 OD, Cal 3 > 0.14 OD, Cal 4 > 0.146 OD.
- Positive control: 363- 957 (660 IU) and negative control: 0–180 (66 IU).

#### Rubella

We used also Euro-immune anti-rubella virus ELISA IgG kits. The content and procedures were as mentioned before in measles.

### Plates are Validated by the Reading of the following:

- Cal 1 > 0.7 (1.839) OD, Cal 2 > 1.178 OD, Cal 3 > 0.361 (0.140) OD, Cal 4 > 0.078 OD.
- Positive control: 29–53 IU and negative control: 0–7 (2 IU).

### Mumps

We used also Euro-immune anti-mumps virus ELISA IgG kits. The kits’ content and preparation were the same as measles, except it has three calibrators and not four as in measles. The procedures were the same as in measles except in the first incubation the serum sample + sample buffer was left together before the following steps for 60 min.

Plates were validated by the reading of the upper limit of normal range of the non-infected individual, at 20 RU/ml, which was recommended by EUROIMMUN as the cut off at < 16 RU/ml is considered negative, at ≥ 16 RU/ml is borderline and at ≥ 22 RU/ml is positive. All measles, mumps and rubella results were calculated quantitative using drawn graphs against optical density.

### Statistical Analysis

Patients’ data were tabulated and processed using SPSS (25.0) statistical package for windows. Quantitative variables were expressed by means and standard deviation and were analyzed using Student’s unpaired t-test.One-way ANOVA test was used to compare more than two groups as regards a quantitative variable (SD > 50% mean). Qualitative data will be expressed by frequency and percent and were analyzed using Chi-square. *P* value > 0.05 = insignificant, *P* value less than 0.05 is insignificant and *P* value less than 0.01 is highly insignificant.

## Results

Although MMR is administered as a triple vaccine, it contains different three virus antigenic structure with different medium, so it is expected to find different immune response to the different three antigen.

Concerning the age within different groups, there was highly significant difference between them except between the ICU and the moderate groups, and as for the relation between the age and the antibodies’ concentration, there was a significant relation in all except measles, which showed high significant positive correlation with the age. Additionally, there was highly significant difference regarding the sex. The relation between age and different age groups is illustrated in Table 1 and the distribution of sex among samples is shown in Table 2.

**Table 1:** Correlation between age (years) and measles, mumps and rubella antibodies titre by Pearson correlation method

Antibodies concentration	Measles	Mumps	Rubella
Age (years)	*r* = 0.153 *p* = 0.041	*r* = 0.188 *p* = 0.011	*r* = 0.536 *p* = 0.001

*r* correlation coefficient, *p* probability significant value

**Table 2:** Distribution of sex difference among different groups

Groups	Control	Mild	Moderate	ICU	Total
Sex					
Male					
Count	31	14	37	36	118
% within group	68.9%	42.4%	63.8%	81.8%	65.6%
Female					
Count	14	19	21	8	62
% within group	31.1%	57.6%	36.2%	18.2%	34.4%
Total					
Count	45	33	58	44	180
% within group	100%	100%	100%	100%	100%

According to the measles’ antibodies concentration level in Table 3, the control group showed statistically significant association with the antibodies level, compared to both the ICU group and the moderate group of patients (*p* < 0.05). Also, the mild group was statistically significant compared to both the ICU and moderate group (*p* < 0.05). A statistically significant association was found between the moderate and ICU group (*p* < 0.05). The antibodies’ level in the ICU group was higher than that of the moderate group. Also, both the ICU and moderate groups have higher antibodies level than the control group.

**Table 3:** Comparative study of measles antibodies concentration by ANNOVA test

Dependent variable	Group A	Group B	Mean difference between Conc. (A-B)	SE	Significance
Measles Conc	Control	Mild	820.278929	384.76165	0.206
		Moderate	–2079.59857	333.50785	0.000
		ICU	–3213.16993	355.93452	0.000

*Conc* Concentration

The antibodies’ level in the ICU group was higher than that of the moderate group, and the relationship was highly statistically significant (*p* < 0.05). The moderate group was highly statistically significant compared to the ICU group (*p* < 0.05). The ICU and moderate groups’ antibodies level was higher than that of the control group, with a high statistical significance. The control group was statistically significant compared to both the ICU and moderate groups (*p* < 0.05). The mild group was statistically significant compared to both the ICU and moderate groups (*p* < 0.05).

According to the mumps antibodies concentration level in Table 4**,** both the control and mild groups presented a statistically significant association with the antibodies level compared to the ICU group (*p* < 0.05) but neither was statistically significant compared to the moderate group or amongst each other (*p* > 0.05). A statistically significant association was found between the moderate and the ICU group (*p* < 0.05). The antibodies’ level in the ICU group was higher than that of the control, mild and moderate groups.

**Table 4:** Comparative study of mumps antibodies concentration by ANNOVA test

Dependent variable	Group A	Group B	Mean difference between Conc. (A-B)	SE	Significance
Mumps Conc	Control	Mild	9.611899	10.007332	1.000
		Moderate	–3.046544	8.674263	1.000
		ICU	–73.509162*	9.257562	0.000

*Conc* Concentration

*The mumps antibodies titre in the ICU group is statistically significant compared to the control, mild and moderate groups (*p* ≤ 0.05), but the other three groups are not statistically significant among each other (*p* > 0.05)

As stated in Table 5 about the rubella antibodies’ concentration level, the control group revealed a statistically significant relation with the antibodies’ level, compared to the moderate, mild and the ICU group (*p* < 0.05). A statistically significant association was demonstrated between the moderate and the ICU group (*p* < 0.05). The mild group showed statistically significant relation compared to the ICU group (*p* < 0.05) but was not- statistically significant when compared to the moderate group (*p* > 0.05). The control group had higher antibodies’ level than the moderate and the mild group but had lower antibodies’ level than the ICU group. The ICU antibodies’ level was higher than that of the moderate group.

**Table 5:** Comparative study of rubella antibodies concentration by ANNOVA test

Dependent variable	Group A	Group B	Mean difference between Conc. (A-B)	SE	Significance
Rubella Conc	Control	Mild	49.377333*	14.049900	0.003
		Moderate	42.869517*	12.178324	0.003
		ICU	–36.573727*	12.997252	0.033

*Conc* Concentration

*Multiple statistically significant associations were found between the control and the mild groups, the control and the moderate groups, the mild and the ICU groups, and the moderate and the ICU groups (*p* ≤ 0.05). A non-statistically significant associations were found between the control and the ICU groups and the mild and the moderate groups (*p* > 0.05)

The multiple relations between the variable groups in the three-virus vaccine (measles, mumps and rubella)-produced antibodies and the different groups of patients are shown in Table 6.

**Table 6:** Illustration of multiple relations between variable groups

Groups	Measles	Mumps	Rubella
Control and mild	Insignificant	Insignificant	Significant = 0.003 Control > Mild
Control and moderate	Significant = 0.000 Moderate > control	Insignificant	Significant = 0.000 Control > moderate
Control and ICU	Significant = 0.000 ICU > control	Significant = 0.000 ICU > Control	Insignificant
Mild and moderate	Significant = 0.000 Moderate > mild	Insignificant	Insignificant
Mild and ICU	Significant = 0.000 ICU > mild	Significant = 0.000 ICU > mild	Significant = 0.000 ICU > mild
Moderate and ICU	Significant = 0.005 ICU > moderate	Significant = 0.000 ICU > moderate	Significant = 0.000 ICU > moderate
Conclusion	Ab. **may not** protect from SARS-CoV-2 infection. Also, it may aggravate the risk of case deterioration	Ab. **may not** protect from SARS-CoV-2 infection. Also, it may aggravate the risk of case deterioration	Ab. **may** protect from SARS-CoV-2 infection, but once infection occurs, it may aggravate the risk of case deterioration

Ab Antibodies

Multiple significant associations were found between multiple groups

## Discussion

COVID-19 is considered a major health problem, with many unclear paradoxes. We need to understand the cause of the wide variation in the virus susceptibility among different individuals with age variation and among different countries. And as the most effective way for protection from various infectious diseases is vaccination, being the most modern technology in the medicine industry, which protects humanity from deadly infections, even after considering its possible side effects or adverse reactions (Segal and Shoenfeld 2018), we aimed in our research to study the association between MMR antibodies and the severity of COVID-19 infection, along with its prognosis.

Many studies were conducted worldwide to correlate MMR antibodies to the susceptibility and severity to COVID-19 infection. In a recent univariate analysis by Cattaruzza et al. (2022), antibodies were found to be inversely associated with COVID-19 disease with non-significant decrease in its infection. In convalescent COVID-19 infected patients from the USA, a high correlation was discovered between T cell responses to S-SARS-CoV-2 and N-SARS-CoV-2 and MMR antibodies in a study by Mysore et al. (2021). While in Iran, a study conducted 2021 suggested that the measles vaccination excited the B cells cross-reactive with SARS-CoV-2 antigens which in turn produced more measles-specific antibody but the mumps and rubella antibodies were found to have no effect (Hassani et al. 2021). An in-silico study funded by the Tunisian Ministry of Higher Education suggested a possible protective effect of different vaccines including the MMR vaccines against COVID-19 (Touati and Elngar 2022).

In our study, we found that the measles and mumps antibodies may not protect from SARS-CoV-2 infection, that they even may increase the risk of autoimmune reaction and lead to case deterioration and admission to the ICU. In refer to the rubella antibodies, while they might protect from initial SARS-CoV-2 infection, but if the infection occurs, they could also increase the risk of case deterioration. So we suggest that MMR antibodies’ titre may be used as a prognostic marker to predict cases, which are susceptible to cytokine storm and which in turn leads to case deterioration.

In contrast to our results and Cattaruzza et al. (2022) results, which showed a direct proportion relationship between COVID-19 severity and the mumps titre, a previous study which reported that only participants with asymptomatic and functionally immune COVID-19 cases had high mumps antibodies titre, and the lower mumps antibodies titre were found in moderate and severe cases. But they could not find the same association between infection with SARS-CoV-2 infection and the rubella antibodies titre. They also did not find any associations between measles antibodies titre and the protection from COVID-19 infection, which agreed with our own findings. They, then concluded that the higher the mumps antibodies titre in a patient, the higher the severity of the COVID-19 infection and that the age was not a factor that affects severity, symptoms or even the antibodies’ titre (Gold et al. 2020).

According to a hypothetical study which was conducted in India, it was reported that children with MMR vaccine released IFNs and NK cells that might in turn protect against infection or ameliorate SARS-CoV-2 (Anbarasu et al. 2020). Another hypothetical Egyptian study suggested that partial protection against COVID-19 infection may occur due to shared structural similarities between measles and SARS-CoV-2, which trigger cross-reactivity in the immune system (Saad and Elsalamony 2020). Partial protection against infectious attacks was suggested by another study also to occur due to the delivery of leukocyte precursors in the bone marrow by trained innate immunity (Fidel and Noverr 2020).

Based upon the lower COVID-19 case report in children than adults, another Indian study explained this phenomenon by the role of structural similarities between SARS-CoV-2 and MMR virus in a child previously immunized against measles (Hanker 2020). Three other hypothetical studies, performed in three different countries, UK, Iraq and USA, concluded that the protection against COVID-19 infection and mortality rate could be reduced due to the MMR immunity (Ashford et al. 2020; Islam et al. 2020; Sidiq et al. 2020). A Mexican study reported that only 16% of the MMR vaccinated population were mildly infected by SARS-CoV-2 (Larenas-Linnemann et al. 2021).

In agreement with our study, the genomic data analysis by Young et al. (2020) discovered a higher IgG rubella level in severe COVID-19 infected patients than in the moderately symptomatic ones. Also, in India, it was reported, in a hypothetical study, that the MMR does not prevent against COVID-19 infection (Deshpande and Balaji 2020).

Taheri Soodejani et al. (2021), concluded in their review article that the majority of the researches which studied the relationship between the MMR immunity and COVID-19 infection were only hypothetical. They, then, suggested performing a clinical trial study for its effectiveness, as well as, its low cost, in comparison to the COVID-19 vaccines.

Based upon Halpert and Shoenfeld (2020) review, they described the SARS-CoV-2 as an autoimmune virus, through the accumulation of data, which enabled the study of SARS-CoV-2 development. It also demonstrated a clear link between this virus infection and auto-immunity, as well as its critical effect on the human immune system. This fact was emphasized by the presence of circulating autoantibodies and autoimmune symptoms in COVID-19 infected patients. Also, the existence of a high mortality rate, caused by the large amount of produced cytokines, resulted in a cytokine storm, large amount of produced ferritin, and fever.

Another study examined autopsies from 18 deceased COVID-19 patients, to see if morphological approaches could reveal autoimmune reactions. They used immunohistochemistry technique on different CD markers on the surface cells, which demonstrated several death mechanisms. They examined the diffused infiltration in different organs and cells, such as lymphocytes to discover their variation among all cases. The CD3^+^ and CD8^+^ markers were dominant in most of the organs, but partly discovered in tissue lesions. Their findings confirmed the autoimmune response caused by the cytotoxic effect of the discovered CD8^+^ (Ehrenfeld et al. 2020).

One of the side effects of vaccines, generally, is the interaction between genetically susceptible healthy individuals and various vaccine components. The adverse reactions are controlled mainly by the molecular mimicry, which leads to cross-reactivity between different vaccine element and specific human proteins causing autoimmune disease (Segal and Shoenfeld 2018).

The innate immunity has evident role in pathogenesis of autoimmune diseases (Abdelsalam et al. 2021). Assuming that antibodies released from previous vaccination, so the second hypothesis depends upon the vaccine may cause misguided trained innate immune response which can contribute to chronic hyperinflammatory state leading to disease progression, organ damage and case deterioration after infection with SARS-CoV-2 virus. Also misguided trained innate immunity may lead to persistent state of immunological tolerance which cause decrease activity of the immune system and subsequent risk of secondary infections and other diseases related to supressed immune system (Netea et al. 2020).

In our study, we measured MMR antibodies but without determining their sources, as it is impossible to know exactly whether they resulted from prior infection or from vaccination. If we supposed that they may be the result of prior infection, then we could link their high antibodies’ titre and autoimmunity cascade (bad prognosis) in COVID-19. Multiple viruses have been associated with autoimmune illnesses, including measles and mumps and rubella. Mumps and rubella infections, in particular, have been associated with the emergence of type-1 diabetes (Ramondetti et al. 2012). Both viruses are capable of infecting and multiplying in beta-cells (Vuorinen et al. 1992). Furthermore, both viruses cause central nervous system demyelination (Virtanen and Jacobson 2012), which is the third hypothesis relative to our results.

The fourth hypothesis is based on the sequence homology of each of the measles, mumps and rubella viruses with SARS-CoV-2 and the direct relationship between the quantity of the viral load and the amount of antibodies in the blood. This similarity between the viruses has been reported by Young et al. (2020), and the homology depends on the similarity between the vaccines, the S-SARS-CoV-2 glycoprotein, the F1-MV and the E1-rubella virus (Fidel and Noverr 2020). This homology may be the cause of the high level of the MMR titre, in response to the high load of SARS-CoV-2 in severe COVID-19 cases, as there is a direct relationship between the viral load and the amount of antibodies in the blood. Wang et al. (2020) reported in their study that the levels of antibodies are higher in the severe cases than the patient with mild symptoms.

It is also important to emphasize that there are ten different mumps strains that have been used in the MMR vaccines worldwide, which lead to different immune response (Wellington and Goa 2003). This may be the fifth cause that supports the hypothesis, as it highlights the difference between our study results and other published research results.

The sixth hypothesis may be explained by antibody-dependent enhancement (ADE) which is considered as an alternative route of viral entry in the susceptible host cell. In this process, MMR antiviral antibodies released by either previous infections or vaccines may enhance the entry access of SARS-CoV-2 virus in the cells by interaction with the complement or Fc receptors leading to the worsening of infection. Data resulted from previous researches on respiratory viruses support the speculation that antibodies elicited against SARS-CoV-2 and during COVID-19 recovery could potentially exacerbate the infection through ADE and this may contribute to disease pathogenesis (Ajmeriya et al. 2022). Therefore, it may explain worsening of cases with high measles and mumps antibodies level.

Notably, COVID-19 is less severe in children due to various factors that may compete the hazards effect of MMR vaccine. Children may have higher levels of melatonin, differences in their microbiota, more frequent recurrent infections which could interfere with the replication of the SARS-CoV-2, differences in innate and adaptive immunity (Zimmermann and Curtis 2021).

In conclusion, it was noted regarding the three viruses; measles, mumps and rubella, there was high antibodies level in the severe COVID-19 infected cases, however, the rubella antibodies were the only one could protect against the SARS-CoV-2 infection, especially at the onset of the disease. The antibodies laboratory test for measles, mumps and rubella viruses, may be used as a good prognostic marker to predict the severity of COVID-19 infection. We also noted that the difference in sex had highly significant difference and a great influence on the severity of the disease as males were the predominant cases in all the infected groups with SARS-CoV-2 with the highest predominance at the ICU group which reached 81.8% of total ICU cases. Also, the age difference had a high significant had high signifcant correlation correlation on SARS-CoV-2 infection.

The limitation of this current work lies in the fact that it is a single study on a relatively small number of patients, so the results may be preliminary. Further multi-centre studies on a larger scale of patients with measuring different cytokines levels (to determine the level of immune activation) and check the presence of antibodies of other respiratory viruses are recommended to prove whether the MMR vaccine is a friend or foe for the COIVD-19 disease.

## Data Availability

The datasets generated during and/or analysed during the current study are available from the corresponding author on reasonable request.

## Code Availability

There is no digital features.
